# Evaluation and Comparison of Antibacterial Efficacy of Herbal Extracts in Combination with Antibiotics on Periodontal pathobionts: An in vitro Microbiological Study

**DOI:** 10.3390/antibiotics8030089

**Published:** 2019-07-01

**Authors:** Shahabe Abullais Saquib, Nabeeh Abdullah AlQahtani, Irfan Ahmad, Mohammed Abdul Kader, Sami Saeed Al Shahrani, Elyas Ali Asiri

**Affiliations:** 1 Periodontics and Community Dental Sciences, College of Dentistry, King Khalid University, Abha 61321, Saudi Arabia; 2 Clinical Laboratory Sciences, College of Applied Medical Sciences, King Khalid University, Abha 61321, Saudi Arabia; 3 Restorative Dental Sciences, College of Dentistry, King Khalid University, Abha 61321, Saudi Arabia; 4 Interns, College of Dentistry, King Khalid University, Abha 61321, Saudi Arabia

**Keywords:** antibacterial activity, antibiotic, periodontal pathobionts, plants extract, synergism

## Abstract

Background: In the past few decades focus of research has been toward herbal medicines because of growing bacterial resistance and side effects of antimicrobial agents. The extract derived from the plants may increase the efficacy of antibiotics when used in combination against pathogenic bacteria. In the current study, the synergistic antibacterial efficacy of plant extracts in combination with antibiotics has been assessed on selected periodontal pathogens. Methods: Ethanolic extracts were prepared from *Salvadora persica* (Miswak) and *Cinnamomum zeylanicum* (Ceylon cinnamon), by the soxhalate method. Plaque samples were collected from clinical periodontitis patients to isolate and grow the periodontal pathobionts under favorable conditions. Susceptibility of bacteria to the extracts was assessed by gauging the diameter of the inhibition zones. Minimum inhibitory concentration (MIC) and minimum bactericidal concentration (MBC) of plant extracts were determined against each bacterium. Synergistic activity of plants extract in combination with antibiotics against the bacteria was also assessed by measuring the diameter of the inhibition zones. Results: Ethanolic extract of both the plants showed an inhibitory effect on the proliferation and growth of all four strains of periodontal pathobionts. Maximum antibacterial activity was exhibited by *C. zeylanicum* against *Tannerella forsythia* (MIC = 1.56 ± 0.24 mg/mL, MBC = 6.25 ± 0.68 mg/mL), whereas among all the studied groups the minimum activity was reported by *C. zeylanicum* against *Aggregatibacter actinomycetemcomitans* the (MIC = 12.5 ± 3.25 mg/mL, MBC = 75 ± 8.23 mg/mL). Combination of herbal extracts with different antibiotics revealed a synergistic antibacterial effect. The best synergism was exhibited by *S. persica* with metronidazole against *A. actinomycetemcomitans* (27 ± 1.78). Conclusions: Current in vitro study showed variable antibacterial activity by experimented herbal extracts against periodontal pathobionts. The synergistic test showed significant antibacterial activity when plant extracts were combined with antibiotics.

## 1. Introduction

Oral and dental health is directly related to systemic health and may be considered as a leading health problem that affects individuals [1]. A large number of infectious diseases persistently represent a source of challenge to the systemic and local health of humans. Oral environment favors for the extended colonization and development of a different variety of microbial species. Diseases of the periodontal structure are considered as one of the most common oral diseases, as old as human society [2]. Periodontitis is an advanced lesion in the supporting periodontal tissue characterized by loss of surrounding alveolar bone, which is considered as one of the main cause of tooth loss in developing and underdeveloped countries [3,4]. 

Longitudinal and cross-sectional studies have confirmed that microbial biofilm and their active by-products are the principal etiology for the gingival and periodontal disease [5,6]. Exotoxins and endotoxins secreted by the periodontal pathobionts play a central role in periodontal disease initiation by destroying the attachment apparatus around the tooth [7]. Out of all bacterial complexes present in biofilm, red complex pathogens are most commonly linked with periodontal disease initiation and progression. The red complex comprises of three different species of bacteria named as; *Porphyromonas gingivalis (P. gingivalis*), *Treponema denticola (T. denticola),* and *Tannerella forsythia (T. forsythia).*

Increased levels of *P. gingivalis*, *T. denticola,* and *T. forsythia* have been detected in stage III and IV periodontitis cases, as well as the red complex (coexistence of all three) species at the same lesion [8,9]. *Aggregatibacter actinomycetemcomitans (A. actinomycetemcomitans),* on the other hand, is frequently related to molar incisor stage IV periodontitis, and selected cases of stage III and IV periodontitis [9,10].

Antimicrobial agents play a vital role in eliminating pathogenic bacteria that invade gingival tissue. In clinical practice amoxicillin, metronidazole, tetracycline, and azithromycin are the most frequently used as adjunctive therapy for the treatment of periodontitis cases [11,12,13]. The exponentially rising multidrug-resistant (MDR) bacteria to present antibiotics is a very critical issue as it represents the predominant cause of treatment failure and increased the percentage of mortality [14]. Thus, it becomes a necessity to develop antibacterial agents that not only prevent the process of drug resistance but also improve the results of the infectious disease treatment.

The rationale for periodontal therapy is to create a “biologically acceptable” root surface by eliminating etiological bacteria and their by-products [15]. Mechanical debridement (consists of scaling and root planing) is the mainstay for prevention and treatment of periodontal disease; in addition to that, chemical plaque control measures can act as adjuvants for maintaining long-term results. The most common and extensively studied chemical plaque control agent is chlorhexidine gluconate. It is considered as the gold standard plaque among control agents because it has most of the features of the ideal antimicrobial solution; such as a broad spectrum of action and substantivity [16]. Studies have revealed that long-term use of chlorhexidine mouthwash is associated with a number of adverse effects like staining of the teeth, loss of taste sensation, degeneration of tongue papilla, and in rare cases parotid swelling [17].

Plants and their extracts are known to be used for therapeutic purpose since the time immemorial, due to the facts that there use is safe, economical, effective, and easily available. *Salvadora persica* (*S. persica*) is a small tree or shrub belongs to the family *Salvadoraceae.* Plant root, stem and twig have been widely used by many people in Africa, the Middle East, and Asian subcontinents for oral hygiene maintenance. It is recommended as an effective tool for oral health care by the World Health Organization (WHO) [18]. Al-Otaibi et al., in randomized control trials, concluded that *S. persica* had a significant effect on reducing the plaque score, gingival inflammation and gingival bleeding [19]. *Cinnamomum zeylanicum* (*C. zeylanicum*), also referred to as ‘true cinnamon’, is an evergreen tree belonging to the *Lauraceae* family, which is native to Sri Lanka. It is obtained from the inner bark of trees. It is commonly used as a food spice in different parts of the world for many centuries. Apart from its traditional use, it has a vital role as a remedy in native Ayurvedic medicine for various types of chronic and infectious diseases. A systemic review on the medical property of cinnamon conducted by Ranasinghe et al. concluded that it has multiple health benefits such as: anti-microbial and anti-parasitic activity, anti-inflammatory activity, wound healing properties, and inhibitory effects on osteoclast [20].

The aim of the present in vitro experimental study was to evaluate the antibacterial effect of *S. Persia* and *C. zeylanicum* plant extract on periodontal pathobionts such as *P. gingivalis, T. denticola, T. forsythia,* and *A. actinomycetemcomitans.* The present study also evaluated the combination potentials of plant extracts with commercially available antibiotics; in other words, to discover new combinations of antimicrobial agents against periodontopathic organisms.

## 2. Materials and Methods

### 2.1. Study Design and Protocol

**Figure 1:**
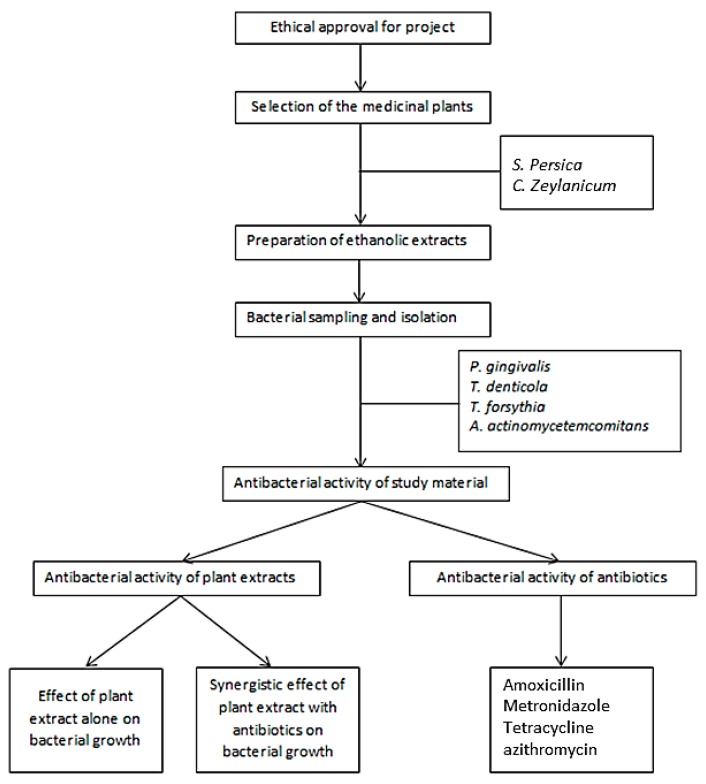
Flow chart of the study design.

An in vitro experimental design was adopted to conduct the study. Ethical clearance was obtained after review and evaluation from the Ethical Review Committee of the institution (King Khalid University, SRC/ETH/2016). Raw plant product (twig of *S. presica* and inner bark of *C. zeylanicum*) was obtained from the herbal garden and native market in Saudi Arabia. Specimens were identified and confirmed by a taxonomist and a pharmacognosist for their authenticity. The plant specimens were submitted to the herbarium of the biology department to obtain the voucher number as follows (*S. presica* #43567 and *C. zeylanicum* #47657). Different antibiotics [metronidazole (lot #34399-42029), amoxicillin (lot #33404-38095), azithromycin (lot #33487-3994), and tetracycline (lot #22363-43393); Research Product International, Mt Prospect, IL, USA] were used in combination with the different plant extracts, to evaluate the existence of a synergistic effects (Figure 1).

### 2.2. Plants Extract Preparation

The raw plant parts were washed with distilled water to remove any unwanted debris and dust. The dust-free parts were dried for 7 days in shade until they were adequately dry to be ground. Each part of the plant was weighed and powdered separately in an electric grinder to obtain a homogenous powder. Soxhalate method was used for the preparation of crude extract. The dried powders were kept in a reciprocating shaker for 72 hours for continuous mixing at a speed of 200 rpm. Ethanol was used as an organic solvent for the extraction purpose. Finally, the crude extracts were filtered by using muslin cloth followed by Whatman no. 1 filter paper. The crude extracts were then stored at −20 °C in a sterile container.

### 2.3. Microbiological Sample Collection

Plaque samples were collected from the deepest pockets around the tooth from the patients diagnosed with active grade III and IV periodontitis for bacterial species (*P. gingivalis*, *T. denticola,* and *T. forsythia*) and molar incisor grade IV periodontitis for bacterial species (*A. actinomycetemcomitans*). The subgingival plaque was collected through inserting Gracey-curette number 5/6 (Hu-Friedy, Chicago, IL, USA) into the periodontal pocket. As soon as curette reaches the base of the pocket without making any injury to the soft tissue, subgingival sampling was performed with one single vertical stroke. The plaque sample was transferred to the paper point #40 taper 0.02 mm/mm (Roeko GmbH Company, Germany) and immediately immersed into the anaerobic transport media [Sodium thioglycolate (Sigma-Aldrich, Taufkirchen, Germany)]. The samples were stored at −80 °C until used for the experimental purpose.

### 2.4. Selective Media for Bacterial Growth

The following specific culture media were used for the growth and isolation of bacteria from the subgingival plaque sample (Table 1). The strains were incubated in an anaerobic chamber (Don Whitley Scientific Ltd., Shipley, West Yorkshire, United Kingdom) with an environment containing 80% nitrogen, 10% hydrogen, and 10% carbon dioxide at 37 °C for 7 days.

### 2.5. Microbiological Assay

#### 2.5.1. Antimicrobial Susceptibility Assays

**Figure 2:**
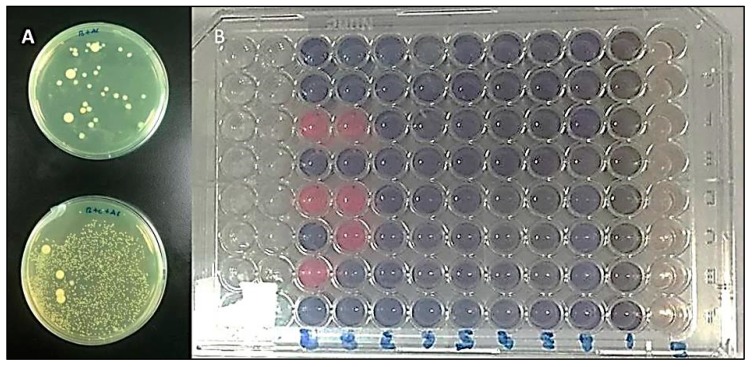
(**A**) Comparison of *T. forsythia* growth in control and after treatment with *C. zeylanicum,* (**B**) Broth microdilution method to evaluate minimum inhibitory concentrations (MIC) and minimum bactericidal concentration (MBC) of plant extract against bacteria.

Agar well diffusion method was used for the antimicrobial activity of the compounds. The bacterial cultures were inoculated in lysogeny broth (LB) media for 3 h at 37 °C and turbidity were adjusted to 0.5 McFarland’s index in phosphate buffered saline. 20 μL of plant extract from different plants (2 mg/mL) was transferred into each well of the Petri-dishes and were incubated anaerobically for 24 h at 37 °C. The diameter of the zone of inhibition of bacterial growth around each well was measured in millimeters as described (Figure 2A) [25].

#### 2.5.2. Determination of Minimum Inhibitory Concentration (MIC)

Micro-broth dilution assays were used to determine minimum inhibitory concentrations (MICs) of the compounds against the bacterial strains. The concentrations of the extracts used for MICs were ranged from 5000 to 50 μg/mL. In brief, the 100 μL culture containing two-fold dilutions of extracts for each strain was loaded in polystyrene sterile flat-bottom 96-well plates duplicate wells (Figure 2B). The starting inoculum for each strain was 1.5 × 10^5^ CFU/mL and the wells containing bacterial inoculum without any extracts were served as a control. The plates were incubated as described above. The lowest concentration of compounds that showed neither visible bacterial growth nor turbidity after 24 hours of incubation in micro-broth dilution assay was considered as MIC. The experiments were repeated in triplicate for each strain.

#### 2.5.3. Determination of Minimum Bactericidal Concentration (MBC)

To determine minimum bactericidal concentration (MBC), 100 μL of the culture from each well of the micro-broth assay was sub-cultured on MH agar plates after 24 hours of the incubation. MH plates were further incubated for 24 hours. The lowest concentration of extracts which exhibited no bacterial growth was deliberated as MBC. The experiments were repeated in triplicate for each strain.

#### 2.5.4. Synergistic Antimicrobial Assays

Antibiotics (azithromycin, tetracycline, metronidazole, and amoxicillin,) were used in combination with plants extract (*S. presica* and *C. zeylanicum*). To determine the synergistic antimicrobial activity, the bacterial strain was spread with a turbidity of 0.5 McFarland on Mueller-Hinton agar (MHA) plates. The discs were anaerobically kept at 37 °C for 24 hours. For the assessments of the synergistic effects, selected antibiotic discs were discretely impregnated with 5 μL of different plant extracts (at the MBC value) and employed on the inoculated agar plates. The zones of inhibition produced by the plant extract in combination with standard antibiotics after overnight incubation were estimated as described [26]: if zones of combination treatment > zone of plant extract + zone of the corresponding antibiotic, was interpreted as synergism; if zone of combination treatment = zone of plant extract + zone of correspondence antibiotic, was interpreted as additive; if zone of combination treatment < zone of plant extract + zone of the corresponding antibiotic, was interpreted as antagonism.

## 3. Results

### 3.1. Antibacterial Activity of Antibiotics and Plant Extracts

**Figure 3:**
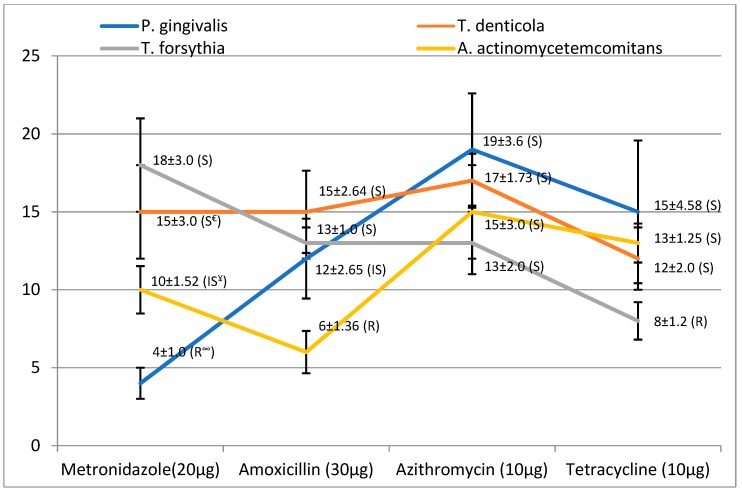
Antibacterial activity of antibiotics against periodontal pathobionts. ^∞^ = Resistant; ^€^ = Sensitive; ^¥^ = Intermediate sensitive.

In the present study, initial screening was done to evaluate the antibacterial activity of selected antibiotics against available bacterial strains. A zone of inhibition measuring more than 8mm signifies that bacteria are susceptible to the tested antibiotics. Figure 3 summarizes the result of antibiotic sensitivity of experimented bacteria against different groups of antibiotic. All the presented bacteria during experimental period showed resistance to at least one antibiotic, except azithromycin, which was active against all the bacteria.

Sensitivity of specific bacteria to specific plant extract was verified by treating selected bacteria to different plant extract. A zone of inhibition measuring more than 8 mm signifies that bacteria are susceptible to the tested plant extract. Table 2 presents the result of antibacterial activity, MIC and MBC exhibited by plant extracts against tested bacteria. All the plant extracts revealed a significant zone of inhibition to the experimented bacteria. Except for *C. zeylanicum* to *A. actinomycetemcomitans* and *S. presica* to *A. actinomycetemcomitans,* showed an intermediate sensitivity.

### 3.2. MIC and MBC of Plant Extracts

MIC and MBC of the plant extracts were determined against all the experimented bacteria. To compare the effect of the different plant extracts on the growth of microorganisms, MIC and MBC of both the plant extracts were a consideration. As the absorption and diffusion of the extract-bioactive compounds that limit the effect on microbial growth in agar medium were ruled out in liquid dilution method used for MIC and MBC determination. Table 3 showed that *C. zeylanicum* and *S. presica* exhibited the highest MIC and MBC against *T. forsythia,* whereas the lowest value for MIC and MBC was presented against *A. actinomycetemcomitans.*

### 3.3. Synergistic Activity of Plant Extracts with Antimicrobial Agents

Synergistic activity of different plant extracts in combination with a number of antibiotics was evaluated by measuring the zone of inhibition method (Table 3 and Table 4). The synergistic activity of individual plant in combination with antibiotics is discussed below;

#### 3.3.1. The Synergy of *C. zeylanicum* with Antibiotics

When *C. zeylanicum* was used in combination with azithromycin, it showed strong synergistic activity against *P. gingivalis* and *T. denticola*. Combination of *C. zeylanicum* with metronidazole and tetracycline showed synergistic outcome against *A. actinomycetemcomitans* and *P. gingivalis,* respectively. The best synergistic combination was observed between *C. zeylanicum* and amoxicillin against the tested bacteria with no antagonism reported (Table 3).

#### 3.3.2. The Synergy of *S. presica* with Antibiotics

Combination of *S. presica* with tetracycline showed a synergistic effect against *P. gingivalis, T. denticola,* and *T. forsythia.* The combination of *S. presica* with amoxicillin revealed a synergistic outcome against *P. gingivalis* and *T. forsythia.* The combination with metronidazole showed a strong synergy only against *A. actinomycetemcomitans* (Table 4). In the present study, *S. presica* presented the best combination with tetracycline against multiple groups of bacteria, in contrast to combination with metronidazole which showed synergy against a single group of bacteria.

## 4. Discussion

The present in vitro experimental study explored the antimicrobial effectiveness of ethanolic plant extract of *S. persica* (Miswak) and *C. zeylanicum* (Ceylon cinnamon) against the target periodontal pathobionts *P. gingivalis*, *T. denticola, T. forsythia,* and *A. actinomycetemcomitans*. In addition, the current study also assessed the synergistic effect of plant extracts in combination with antibiotics against periodontal pathogens.

The primary objective of the field of ethnopharmacology is to identify and develop efficient and accessible medication from active plant compounds having minimal side effects. Additionally, active compounds from the plant extract with antibacterial activity can be transformed into possible medication. These medications can be used as therapeutic agents for the prevention and control of infectious diseases [27].

Antimicrobial therapy is considered as the adjunctive to conventional periodontal therapy for the management of periodontal diseases [28]. However, despite optimum periodontal therapy some of the individuals continued to show attachment loss may be because of the invasion of pathogenic bacteria [29]. In such cases, adjunctive use of systemic antibiotics is emphasized to support the host defense mechanism in combating against the pathogenic microorganisms. The antibiotics most commonly used as an adjunct to periodontal therapy are; metronidazole, clindamycin, amoxicillin, clavulanate, azithromycin, and tetracycline [29,30,31,32]. Thus, in the present study, we had selected the group of antibiotics (metronidazole, amoxicillin, azithromycin, and tetracycline) that are commonly used to treat periodontal infections.

Based on the findings from the past, in the current experiment ethanol has been selected as a solvent to extract active compounds from the plant products [33,34]. Findings from previous phytochemical analysis revealed that *C. zeylanicum* (bark) contains active ingredients like; alkaloid, flavonoid, steroid/triterpenoid, tannin, and quinone [35]. These compounds are known to have antibacterial, anti-inflammatory, and antifungal properties. Extracts of *C. zeylanicum* are known to exhibit a wide antibacterial spectrum against gram positive and gram negative bacteria, with higher activity against gram-negative [36]. There are various in vitro and in vivo studies available in the literature evaluating the efficiency of *C. zeylanicum* essential oils against several oral microorganisms. Most of these studies have been done on the cariogenic bacteria and primary colonizer of plaque, and the results revealed that these bacteria are sensitive to the oils of *C. zeylanicum* [37,38]. In the recent past, a study conducted by Zainal-Abidin et al. showed the significant antibacterial effect of cinnamon oil against oral pathogens viz. *S. mutans, S. mitis, S. salivarius, A. actinomycetemcomitans, P. gingivalis,* and *F. nucleatum.* [38]. Another microbiological study conducted by Kim et al. reported that ethanolic extract of cinnamon was effective in suppressing the acid production and bacterial adhesion for cariogenic bacteria [33]. There is still a scarcity of the studies evaluating the antibacterial effect of ethanolic extract from *C. zeylanicum* on periodontal pathobionts. To the best of authors’ knowledge, there is no reported study which evaluates the in vitro effect of *C. zeylanicum* on periodontal pathobionts. The result of the present study demonstrated the antibacterial activity of *C. zeylanicum* against all the tested periodontal pathobionts. The antibacterial activity was highest against *T. forsythia* (MIC = 1.56 ± 0.24 mg/mL, MBC = 6.25 ± 0.68 mg/mL), and lowest against *A. actinomycetemcomitans* (MIC = 12.5 ± 3.25 mg/mL, MBC = 75 ± 8.23 mg/mL). The results of present study are in agreement with the findings of the study conducted by Aneja et al., reported significant antibacterial activity of acetone, ethanol and methanol extracts of *C. zeylanicum* against oral bacteria and yeast [39].

*S. persica* is commonly known as miswak and it is used as an oral hygiene tool by many people. The oral health care is achieved by dual mechanism, mechanically by the presence of multiple fibers, whereas chemically by the presence of phytochemical constituents. Reports from the previous investigations showed that it has significant antibacterial, anti-inflammatory and remineralization activity without any reported toxicity [40,41]. Chemical compounds isolated from plant parts contain tri-methyamin, salvadorine, chloride, fluoride, silica, sulfur, vitamin C, saponins, tannins, and benzyl-isothiocyanate. Clinical studies from the recent past have demonstrated the antigingivitis and antiplaque effect of the chewing stick, and when used as a mouth wash it showed improvement in the gingival and periodontal health by reducing the bacterial load and lowering the carriage rate of pathogenic bacteria [42,43]. Various solvents such as water, ethanol, methanol, hexane, and acetic acid have been used in the past for the extraction of a bioactive compound from the different parts of the plant. Based on the facts form majority of experimental studies, alcoholic extract (particularly ethanolic) exhibits maximum antibacterial activity as compared to other extracts [44,45]. Many studies are available in the literature presenting the effect of *S. persica* extract on the large range of oral and general bacteria, but its effect on particular periodontal pathogens has not been studied extensively. In the present study, the extract of *S. persica* showed antibacterial activity against all the studied periodontal pathobionts. The highest antibacterial activity was reported against *T.forsythia* (MIC = 3.12 ± 0.85 mg/mL, MBC = 11.5 ± 3.21 mg/mL) followed by *T. denticola* (MIC = 6.25 ± 1.15 mg/mL, MBC = 25 ± 3.25 mg/mL)*, P. gingivalis* (MIC = 6.25 ± 1.24 mg/mL, MBC = 50 ± 6.21 mg/mL), and minimum against *A. actinomycetemcomitans* (MIC = 12.5 ± 2.25 mg/mL, MBC = 50 ± 6.35 mg/mL). The findings from the present study are in agreement with the results of the study conducted by Sofrata et al, in which they found a strong inhibitory effect of *S. persica* extract on inhibitory effect on *P. gingivalis* and *A. actinomycetemcomitans*. The 0.14-g suspended *S. persica* in agar showed greater inhibition on A. actinomycetemcomitans [46]. In another, in vitro study authors evaluated the effect of alcoholic extract of *S. persica* against bacterial strains isolated from the saliva of periodontitis patients, and they conclude that extract obtained from *S. persica* is active against all the isolated bacterial strains [47].

Antibacterial spectrum of the available antibiotics can be magnified by the combination of phytopharmaceuticals and modern medication. In addition, the combination may be helpful in the preclusion of the emergence of resistant bacteria and reducing the drug toxicity. The outcome of combining two drugs can be synergistic, additive, or antagonistic depending on the interaction between drugs. Various in vitro experiments have established the fact that a combination of plant extracts and antibiotics possess a synergistic effect, which results in a significant decrease in levels of MIC for the antibiotics [48,49]. One of the main objectives of the current study was to estimate and establish the combined antibacterial activity of plant extracts with antibiotics against clinical strains of periodontal disease causing bacteria. The findings from the present study may help to understand the synergistic effect of combination therapy, which in the recent future could provide a new strategy for the adjunctive treatment for periodontal infections. Results from the current study revealed that a combination of *C. zeylanicum* with azithromycin revealed synergistic activity against *P. gingivalis* and *T. denticola,* whereas combination with amoxicillin showed synergism against *T. denticola* and *T. forsythia* (strongest synergism = 38 ± 4.12 mm)*. A. actinomycetemcomitans* and *P. gingivalis* showed increased sensitivity to metronidazole and tetracycline respectively when used in combination with *C. zeylanicum. S. presica,* when used in combination with tetracycline, showed synergistic antibacterial activity against all the studied periodontal pathobionts except *A. actinomycetemcomitans.* This combination showed synergistic activity against a maximum number of bacteria tested in the current study. The combination of *S. presica* with amoxicillin showed synergism against *P. gingivalis* and *T. forsythia. A. actinomycetemcomitans* revealed higher sensitivity after treatment with a combination of *S. presica* with metronidazole and azithromycin, respectively. The above-discussed findings are in accordance with the previous study, in which the author had reported synergism between ethanol plant extract and gentamycin against *P. gingivalis, T. denticola, T. forsythia* [50]. A similar study in the recent past also concluded that plant extracts enhanced the activity of tetracycline two- to four-fold, against resistant strains of periodontal bacteria [51].

## 5. Conclusions

Emerging bacterial resistance is a major concern for the medical field, despite the fact that pharmaceutical companies have synthesized a large number of antibiotics in the recent past. Results from the ethnopharmacological studies suggest that the plants and their products are a good source of biologically active antibacterial agents. This activity can be enhanced by the synergism between herbal extracts and known antibiotics which could offer significant potential for the development of novel antimicrobial therapeutic agents. Findings from the current in vitro study revealed that ethanolic extracts from *C. zeylanicum* and *S. persica* plants exhibit antibacterial activity against all the experimented periodontal pathobionts. The synergistic test results showed significant antibacterial effects when plant extracts were combined with the antibiotics. Combination of *S. presica* with tetracycline showed synergistic antibacterial activity against maximum periodontal pathobionts, and the strongest synergism was reported between *C. zeylanicum* and amoxicillin against *T. denticola.* However, further research is required to understand the mechanisms involved in the synergistic activity. Only with a proper understanding of these mechanisms will it be possible to form standardized and effective preparation against periodontal pathobionts.

## Figures and Tables

**Table 1:** Specific media used for bacterial growth.

Bacteria	Media
*P. gingivalis*	(Columbia agar base *, Bacitracin *, Colistin *, Nalidixic acid *) [21]
*A. actinomycetemcomitans*	(Trypticase soy *, Bacitracin *, Vancomycin ** (TSBV)) [22]
*T. forsythia*	(Tryptic soy broth *, Yeast extract **, Vit. K *, N-Acetylmuramic acid *) [23]
*T. denticola*	(Oral bacteria growth medium (OBGM)) [24]

* = Sigma Aldrich Co Ltd, St. Louis, MO, USA; ** = HiMedia Laboratories Pvt. Ltd, Mumbai-86, India.

**Table 2:** Antibacterial activity, MIC and MBC exhibited by ethanolic plant extracts against periodontal pathobionts.

Bacteria	Ethanolic Extract					
	*C. zeylanicum*			*S. presica*		
	Zone (mm) (mean ± SD)	MIC (mg/mL) (mean ± SD)	MBC (mg/mL) (mean ± SD)	Zone (mm) (mean ± SD)	MIC (mg/mL) (mean ± SD)	MBC (mg/mL) (mean ± SD)
*P. gingivalis*	18 ± 1.5	3.12 ± 0.65	12.5 ± 1.35	15 ± 0.35	6.25 ± 1.24	50 ± 6.21
*T. denticola*	13 ± 1.0	6.25 ± 1.24	50 ± 5.24	14 ± 1.75	6.25 ± 1.15	25 ± 3.25
*T. forsythia*	21 ± 1.75	1.56 ± 0.24	6.25 ± 0.68	19 ± 1.56	3.12 ± 0.85	11.5 ± 3.21
*A. actinomycetemcomitans*	8 ± 0.75	12.5 ± 3.25	75 ± 8.23	10 ± 2.0	12.5 ± 2.25	50 ± 6.35

**Table 3:** Synergistic antimicrobial activity of *C. zeylanicum* with different antimicrobial agents.

Bacteria	Antibiotics (anti.)	MIZ * with anti. (mm) (mean ± SD)	MIZ with *C. zeylanicum* (mm) (mean ± SD)	MIZ with anti. and *C. zeylanicum* (mm) (mean ± SD)	Outcome
*P. gingivalis*	Metronidazole	4 ± 1.0	18 ± 1.5	20 ± 3.25	Antagonism
	Amoxicillin	12 ± 2.65		30 ± 4.12	Additive
	Azithromycin	19 ± 3.6		40 ± 4.85	Synergism
	Tetracycline	15 ± 4.58		36 ± 4.02	Synergism
*T. denticola*	Metronidazole	15 ± 3.0	13 ± 1.0	27 ± 3.21	Antagonism
	Amoxicillin	15 ± 2.64		30 ± 3.65	Synergism
	Azithromycin	17 ± 1.73		34 ± 3.75	Synergism
	Tetracycline	12 ± 2.0		21 ± 2.85	Antagonism
*T. forsythia*	Metronidazole	18 ± 3.0	21 ± 1.75	34 ± 4.01	Antagonism
	Amoxicillin	13 ± 1.0		38 ± 4.12	Synergism
	Azithromycin	13 ± 2.0		30 ± 3.12	Antagonism
	Tetracycline	8 ± 1.2		29 ± 3.04	Additive
*A.* *actinomycetemcomitans*	Metronidazole	10 ± 1.52	8 ± 1.75	20 ± 2.26	Synergism
	Amoxicillin	6 ± 1.36		14 ± 1.96	Additive
	Azithromycin	15 ± 3.0		21 ± 3.05	Antagonism
	Tetracycline	13 ± 1.25		18 ± 2.85	Antagonism

* MIZ: Mean of inhibition zone.

**Table 4:** Synergistic antimicrobial activity of *S. presica* with different antimicrobial agents.

Bacteria	Antibiotics (anti.)	MIZ * with anti. (mm) (mean ± SD)	MIZ with *S. presica* (mm) (mean ± SD)	MIZ with anti. and *S. presica* (mm) (mean ± SD)	Outcome
*P. gingivalis*	Metronidazole	4 ± 1.0	15 ± 0.35	18 ± 1.68	Antagonism
	Amoxicillin Azithromycin Tetracycline	12 ± 2.65 19 ± 3.6 15 ± 4.58		31 ± 2.15 34 ± 3.5 33 ± 3.78	Synergism Additive Synergism
*T. denticola*	Metronidazole	15 ± 3.0	14 ± 1.75	29 ± 2.69	Additive
	Amoxicillin	15 ± 2.64		27 ± 1.45	Antagonism
	Azithromycin	17 ± 1.73		31 ± 2.65	Additive
	Tetracycline	12 ± 2.0		28 ± 2.96	Synergism
*T. forsythia*	Metronidazole	18 ± 3.0	19 ± 1.56	32 ± 3.02	Antagonism
	Amoxicillin	13 ± 1.0		37 ± 3.47	Synergism
	Azithromycin	13 ± 2.0		32 ± 4.02	Additive
	Tetracycline	8 ± 1.2		31 ± 2.58	Synergism
*A.* *actinomycetemcomitans*	Metronidazole	10 ± 1.52	10 ± 2.0	27 ± 1.78	Synergism
	Amoxicillin	6 ± 1.36		14 ± 0.85	Antagonism
	Azithromycin	15 ± 3.0		28 ± 3.26	Synergism
	Tetracycline	13 ± 1.25		22 ± 1.52	Antagonism

* MIZ: Mean of inhibition zone.
